# Stabilization of Traumatic Iliosacral Instability Using Innovative Implants: A Biomechanical Comparison

**DOI:** 10.3390/jcm13010194

**Published:** 2023-12-29

**Authors:** Niklas Grüneweller, Julia Leunig, Ivan Zderic, Boyko Gueorguiev, Dirk Wähnert, Thomas Vordemvenne

**Affiliations:** 1 Bielefeld University, Medical School and University Medical Center OWL, Protestant Hospital of the Bethel Foundation, Department of Trauma and Orthopedic Surgery, Burgsteig 13, 33617 Bielefeld, Germanydirk.waehnert@evkb.de (D.W.); 2 AO Research Institute Davos, Clavadelerstrasse 8, 7270 Davos, Switzerland; julialeunig@icloud.com (J.L.); ivan.zderic@aofoundation.org (I.Z.);

**Keywords:** dorsal pelvic ring, biomechanic, SI-plate, triangular fixation, iliosacral instability

## Abstract

(1) Background: Demographic changes over the past decade have had a significant impact on pelvic ring fractures. They have increased dramatically in the orthogeriatric population. Surgeons are faced with implant fixation issues in the treatment of these fragility fractures. This study compares two innovative implants for stabilizing the iliosacral joint in a biomechanical setting. (2) Methods: An iliosacral screw with a preassembled plate allowing the placement of an additional short, angular stable screw in the ilium and a triangular fixation system consisting of a fenestrated ilium screw and an iliosacral screw quasi-statically inserted through the “fenestra” were instrumented in osteoporotic artificial bone models with a simulated Denis zone 1 fracture. Biomechanical testing was performed on a servo-hydraulic testing machine using increasing, synchronous axial and torsional sinusoidal cyclic loading to failure. (3) Results: The SI-Plate and TriFix showed comparable stiffness values. The values for fracture gap angle and screw tip cutout were significantly lower for the TriFix compared to the SI-Plate. In addition, the number of cycles to failure was significantly higher for the TriFix. (4) Conclusions: Implant anchorage and primary stability can be improved in iliosacral instability using the triangular stabilization system.

## 1. Introduction

Fractures of the posterior pelvic ring are a major issue in trauma and orthogeriatric surgery. In the last few decades, the epidemiology of these injuries has changed considerably.

A recent analysis of the German Pelvic Trauma Registry showed that women are more often affected by pelvic fractures than men (incidence of 33.4/100,000 for men; 38.4/100,000 for women) [1]. In particular, the number of orthogeriatric patients suffering from pelvic fractures is increasing rapidly [2,3]. As a result, the majority of pelvic fractures today occur in elderly patients [4]. But it is not only the age and sex distribution of pelvic fractures that has changed. Fracture morphology has also changed dramatically. While the incidence of type A fractures decreased substantially (from 85% in 1991 to 44% in 2013), the incidence of type C fractures (from 7% in 1991 to 14% in 2013) and especially type B fractures (from 8% in 1991 to 42% in 2013) increased significantly [3].

Pelvic fractures, especially in the elderly, are very different from high-energy fractures in terms of symptoms and treatment. In the face of these dramatic demographic changes, the management of older patients is becoming increasingly important. The specific challenges of treating elderly patients include existing comorbidities, lack of physical fitness, and mental health conditions such as dementia [5]. In addition, reduced bone quality in this population is another major factor that makes it difficult to adequately treat patients with fragility fractures of the pelvis [6]. A classification system was developed by Rommens and Hofmann to address the specific needs of patients suffering from these fragility fractures [2]. Many patients have comorbidities that put them at risk of complications and increased mortality [6]. As a result, there is no consensus on the indications for and type of surgical treatment of pelvic fragility fractures [5]. Both surgical and conservative treatment options have their benefits and risks. While conservative treatment puts patients at risk of pneumonia and urinary tract infection due to immobilization, operative treatment is associated with surgical complications such as hematoma and surgical site infection. In addition, the fragile bone increases the risk of further collapse with conservative treatment and implant loosening with surgical treatment [5].

Pain relief and early mobilization are the main goals in the treatment of fragility fractures of the pelvis. Any treatment should, therefore, be less invasive, aim to improve general health, and prevent further fragility fractures [7].

As a result, iliosacral screw osteosynthesis is now a well-established technique and is still considered the standard of care for many patients with fractures of the dorsal pelvic ring. This type of treatment is minimally invasive, provides adequate pain relief, and allows patient mobilization immediately after surgery [5,8]. A major disadvantage of this procedure is the reduced anchorage of the implant in the porous bone with the risk of screw loosening [9,10].

Several modifications aimed at increasing implant fixation have been introduced to address this major problem. Screw tip augmentation and screw-in-screw prototypes are two of these innovations [11,12,13,14,15,16]. In biomechanical comparisons, augmentation and screw-in-screw techniques have been shown to increase stability in osteoporotic bone and to prevent certain failure mechanisms, namely screw back-out [13,17].

The design and manufacture of new implants, especially those with connected parts, is more difficult and must take into account several aspects. It is well known that the implant material is crucial for bone-implant-load interaction (e.g., stress shielding) and can have an effect on tribocorrosion in connected implants [18]. Titanium alloys are widely used for orthopedic implants due to their superior strength-to-weight ratio, biocompatibility, and corrosion resistance. However, Ti-6Al-4V, in particular, does not have inherent tribocorrosion resistance. Therefore, it is necessary to eliminate or minimize metal-to-metal contact in motion areas. In pelvic implants, the washer head is such an area where minimal motion could occur. Due to the small contact area (screw head and washer) and the minimal motion, no clinical problems, such as aseptic loosening, have been reported. Implant areas with higher expected motion and larger contact areas such as a screw-in-screw (e.g., fenestrated iliac screw with iliosacral screw) have a higher potential for this problem. Measures such as polyethylene inlays have a dual effect: they reduce tribocorrosion by minimizing the metal-to-metal contact as well as by reducing motion.

The aim of this study was to compare the biomechanics of two implant configurations of an innovative modular implant system for stabilizing the dorsal pelvic ring. Two groups were compared in an artificial pelvis model. An iliosacral screw (Silony Medical AG, Frauenfeld, Switzerland) offering a double-threaded pedicle screw design for rapid insertion and improved primary stability with a pre-mounted plate (corresponding to an enlarged washer) and an additional angular stable plate screw was compared to a construct combining a uniquely designed fenestrated iliac screw (Silony Medical AG, Frauenfeld, Switzerland) with the above mentioned iliosacral screw providing an angular stable construct for the dorsal pelvic ring.

## 2. Materials and Methods

### 2.1. Implants

**Figure 1:**
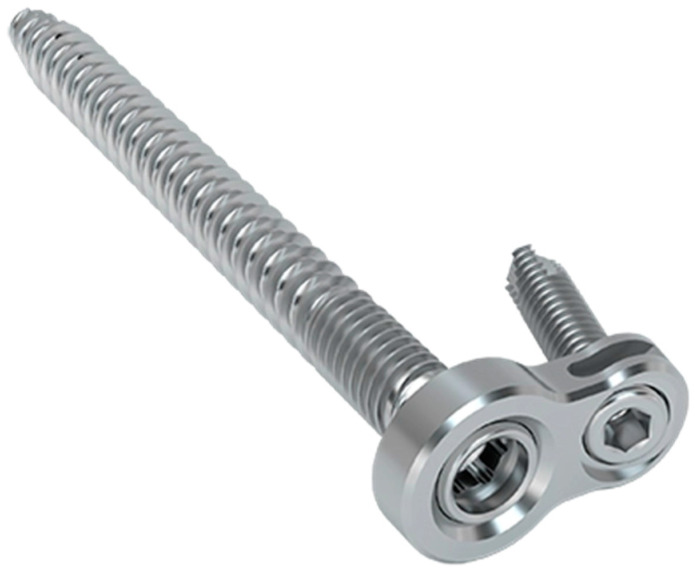
Picture of the SI-Plate, consisting of the double-threaded iliosacral screw with a pre-mounted plate that acts as a washer and provides the option of placing a short, angular stable screw for fixation in the ilium.

In this study, we used two different percutaneous implant configurations to stabilize the posterior pelvic ring. All implants were made from Ti6Al4V ELI, a well-known and widely used material for medical implants. Group I was stabilized with a 7.2 mm iliosacral screw with a pre-mounted plate (SI-plate, Silony Medical GmbH, Frauenfeld, Switzerland). The plate allowed the placement of an additional short angular stable screw in the ilium (Figure 1). The iliosacral screw used for this study had a length of 100 mm. The angular stable locking was a 3.5 mm screw with a length of 20 mm. This iliosacral screw has been designed with biomedical needs in mind. Therefore, the double thread design is used to allow rapid insertion combined with good primary stability through some interfragmentary compression. The pre-mounted plate with the option of inserting a short, angular stable screw into the ilium secures the construct against unthreading and increases primary stability. 

**Figure 2:**
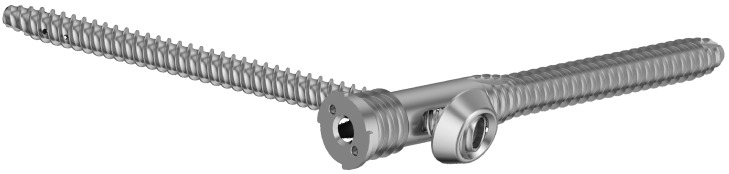
Image of the TriFix implant showing a fenestrated iliac screw and an iliosacral screw with a pre-mounted washer. This configuration provides an almost angular stable connection between the ilium and the iliosacral screw.

Group II was stabilized with the triangular fixation system (TriFix, Silony Medical GmbH, Frauenfeld, Switzerland). This system consists of a fenestrated iliac screw with a 9.2 mm diameter anterior screw portion and a 14 mm diameter fenestrated portion, and an iliosacral screw with a pre-mounted washer (Figure 2). The iliosacral screw is inserted through the “fenestra” of the ilium screw by using an aiming arm device. Due to a polyethylene inlay in the “fenestra”, quasi-angular stable fixation is provided. The TriFix design allows stepwise and modular surgical treatment of the dorsal, pelvic ring according to the biomechanical needs of the fracture or instability. The primary stability of the construct is increased by the quasi-angle stable connection of the iliac screw and the iliosacral screw in combination with the additional medial support of the iliosacral screw. As mentioned above, the modular design allows for easy extension to spinopelvine stabilization.

Actually, no connection between the pelvic ring and the lumbar spine was established in this study, but still, this iliac screw is referred to as the “TriFix” screw. 

### 2.2. Bone Model

Sixteen artificial pelvises with simulated osteoporotic bone structure (LS4060, Synbone AG, Zizers, Switzerland) were used for this investigation. This bone model has already been successfully used in several biomechanical studies on the posterior pelvic ring [11,13].

### 2.3. Fracture Model and Instrumentation

On the right sacral side of each model, a vertical paraforaminal osteotomy was performed in Denis classification zone 1 using a band saw. A custom-made cutting guide was used to achieve consistent fracture lines. The symphysis and the left sacroiliac joint were then cut wide to disrupt the pelvic ring. The left hip bone was excluded from further use in this study [13]. The specimens were randomly assigned for instrumentation with an iliosacral screw plus locking screw (SI-plate) in Group I or with an iliosacral screw through a fenestrated ilium screw (TriFix) in Group II. 

The SI joint was rigidly fixed in both groups using wood screws to simulate an ossified and fused joint, a common scenario in the elderly, and to concentrate the forces acting on the sacral fracture. The posterior pelvic ring fragments were anatomically reduced and instrumented in a standardized manner using custom-made drill guides to ensure standardized screw placement in each specimen for both groups. Instrumentation was carried out using the appropriate manufacturers’ instruments and in accordance with the manufacturers’ instructions. All specimens were instrumented by one experienced pelvic surgeon. 

**Figure 3:**
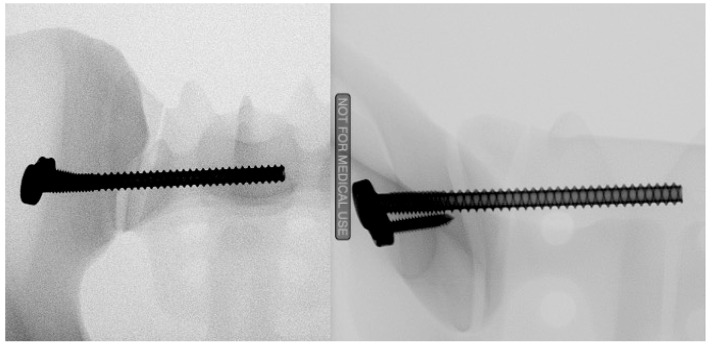
Radiograph of the SI plate fixation in two planes.

For the SI plate fixation in Group 1 (Figure 3), using a drill guide, a 3.2 mm guidewire was first inserted across the SI joint into the first sacral body under radiographic control. The guide wire was then over-drilled, followed by the insertion of a 100 mm long 7.2 mm fully threaded self-cutting cannulated SI with a pre-mounted plate. The screw was tightened according to the surgeons’ best practice. The orientation of the plate was standardized posteriorly (9 o’clock orientation). After SI screw placement, the hole for the short-locking screw was prepared by drilling a 2.0 mm hole over the drill sleeve. A 20 mm head locking screw was then inserted and tightened at 4 Nm using a torque limiter.

**Figure 4:**
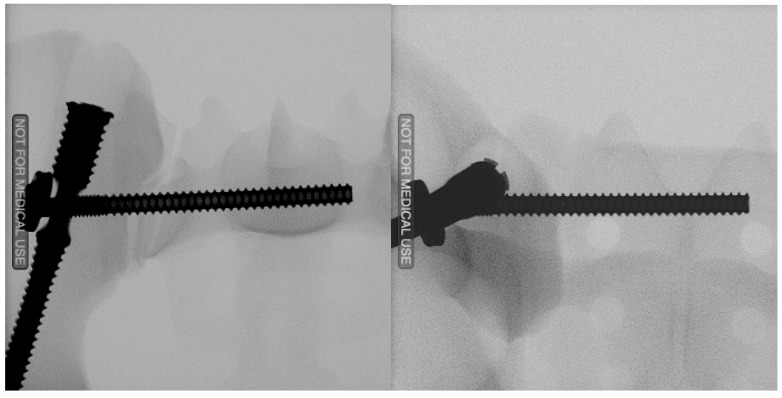
Radiograph of the TriFix fixation in two planes.

In Group 2 (Figure 4), the TriFix instrumentation began with the insertion of the iliac screw. A 3.2 mm guide wire was placed over the custom-made drill guide and inserted into the ilium under radiographic guidance from the posterior iliac spina. After correct wire placement, the screw hole was prepared by drilling and thread cutting. Afterward, the iliac screw was inserted over the guide wire to the correct depth. After that, an aiming device was mounted, allowing to interlock the ilium screw with the iliosacral screw. Afterward, the wire for the sacroiliac screw was placed using the mounted aiming arm and radiographic control. Once the correct position was achieved, the wire was over-drilled, and the 100 mm long 7.2 mm iliosacral screw was inserted. No additional locking screw was inserted into the sacroiliac screw plate in this case.

### 2.4. Biomechanical Testing

Biomechanical testing was performed on a biaxial servo-hydraulic testing machine (MTS 858 MiniBionix, MTS Systems Corp, Eden Prairie, MN, USA) equipped with a 5 kN/50 Nm load cell. The setup was adopted from previous studies [13]. Pre-tests were conducted to achieve a clinically relevant failure mode. Therefore, a muscular preload had to be included to prevent the pelvis from bending.

**Figure 5:**
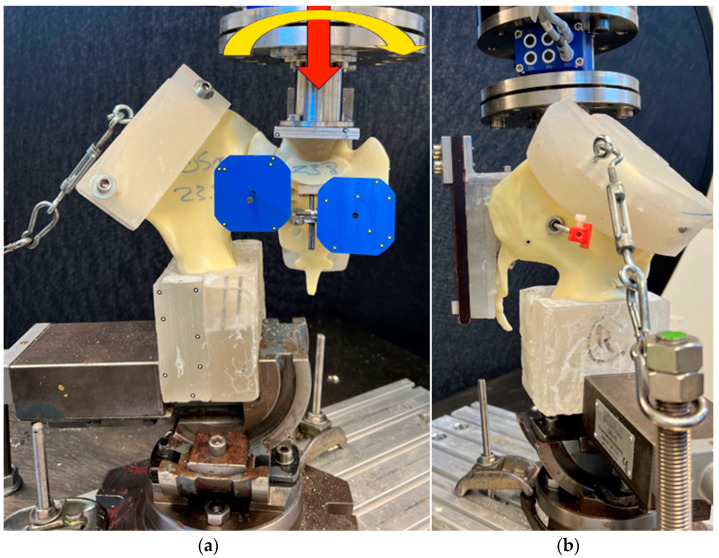
Test setup with a specimen mounted for biomechanical testing with colored arrows visualizing the loading directions. View from (**a**) anterior and (**b**) lateral.

Each specimen was aligned in an upright standing position with its distal portion secured to the machine base using a vice and X-Y table (Figure 5). The latter facilitated the mounting of the specimen by mediolateral and anteroposterior sliding and was clamped in place during the test.

The proximal part of the specimen was attached to the load cell and machine actuator via an L-shaped frame, which was secured to the posterior aspect of the sacrum with screws through the foramina. Muscle tension was simulated via a turnbuckle connecting the machine base with the iliac crest. For that purpose, a PMMA block was attached to the iliac crest and served as an anchor for the turnbuckle. Optical markers were attached to the sacrum medial and lateral to the fracture and to the iliosacral screw.

A muscular preload of 15 N was applied prior to biomechanical testing. The loading protocol commenced with a quasi-static axial compression ramp from 15 N to 100 N at a rate of 8.5 N/s, followed by synchronous axial and torsional sinusoidal cyclic loading to failure at 2 Hz. During the cyclic test, the axial load was progressively increased at a rate of 0.05 N/cycle from its initial peak value of 100 N. Torsional loading started at 0.5 Nm in external rotation with an increment of 0.00025 Nm/cycle. Test stop criteria were set at 30 mm actuator displacement with respect to its position at test start. 

### 2.5. Data Evaluation and Statistics

Machine data in terms of axial displacement (mm) and axial load (N), as well as torsional angle (°) and torque (Nm), were recorded from the machine controllers at 128 Hz. The initial stiffness was calculated from the rising slope of the load-displacement curve of the quasi-static test ramp within a load range of 30–60 N.

Two optical cameras (Aramis SRX, GOM GmbH, Braunschweig, Germany) continuously recorded the marker positions at 50 Hz for motion tracking, with a resolution of 12 megapixels and a maximum acceptance error of 0.004 mm. Based on the motion tracking data, the fracture gap opening between the two initially reduced osteotomy surfaces of the medial and lateral sacral fragments relative to each other was calculated as a combined rotational movement in the coronal and transverse plane and defined as a gap angle. In addition, the movement of the SI-screw tip perpendicular to its axis within the sacrum was calculated as the screw tip cutout. The margins of these two parameters were evaluated at three time points after 2000, 4000, and 6000 cycles with respect to the corresponding values at the third test cycle to consider specimens’ settling. A screw tip cutout of 2 mm was defined as the failure criterion, and the corresponding numbers of cycles until its fulfillment were calculated together with the corresponding load. All evaluations were performed under peak axial compressive loading. The evaluation algorithm was based on the publication of Zderic et al. [13].

Radiographs were taken in the anteroposterior direction at the beginning of the cyclic test and then every 500 cycles using a triggered C-arm (Siemens ARCADIS Varic, Siemens Medical Solutions AG, Erlangen, Germany) to determine the point of failure of the screw fixation and to investigate its mechanism.

Statistical analysis among the parameters of interest was performed using SPSS software (version 23, IBM SPSS, Chicago, IL, USA). The mean and standard deviation were calculated for each parameter of interest. Independent-sample t-tests and three-way General Linear Model (GLM) Repeated Measures (RM) tests were performed to detect significant differences between the two study groups for cross-sectional (initial stiffness, cycles to failure) and longitudinal (values at 2000, 4000, and 6000 cycles) data, respectively. *p* values < 0.05 were considered significant.

## 3. Results

### 3.1. Stiffness

The mean initial construct stiffness was 62.6 N/mm (SD 20.3 N/mm) for the SI-plate group and 49.7 N/mm (SD 17.1 N/mm) for the TriFix group. This difference of approximately 26% was statistically not significant (*p* = 0.245). 

### 3.2. Fracture Gap-Angle and Screw Tip Cutout

**Figure 6:**
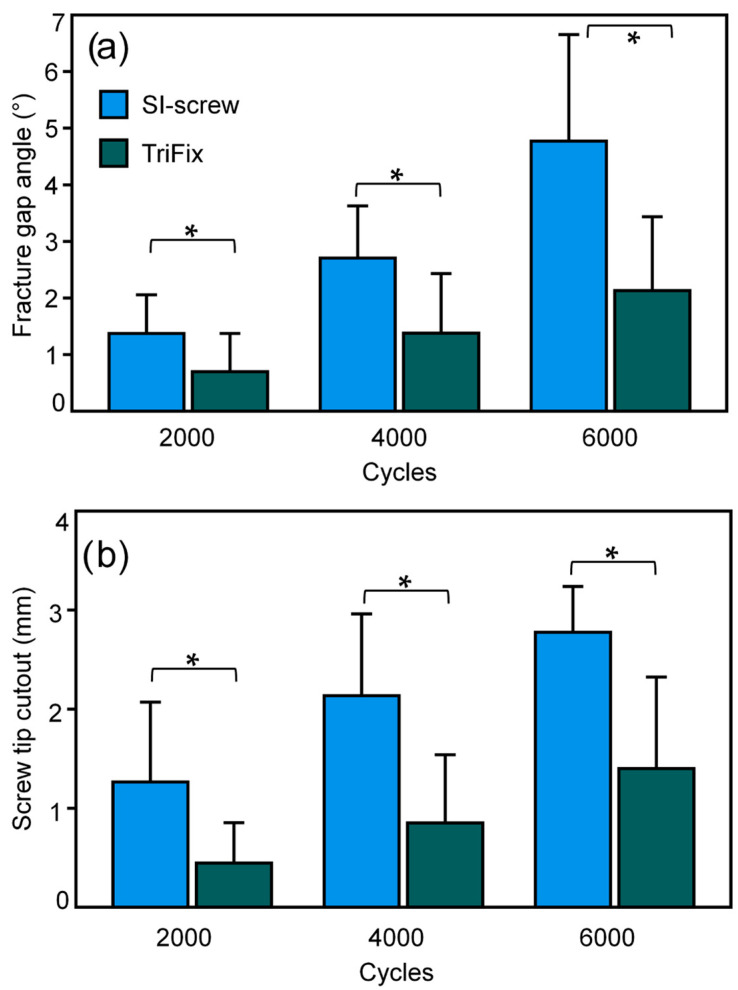
Fracture gap-angle (**a**) and screw tip cutout (**b**) are shown at intermittent time points after 2000, 4000, and 6000 cycles for each group separately in terms of mean and SD. Significant differences between the groups are marked with *.

Figure 6 shows the mean values for the two parameters evaluated over the first 6000 cycles at three intermittent time points, namely fracture gap angle and screw tip cutout, for both groups separately. For both parameters, the TriFix was associated with significantly lower values compared to the SI-plate (*p* = 0.019/0.011). The difference for the fracture gap angle was +72% at 2000 cycles, +71% at 4000 cycles, and +98% at 6000 cycles for the SI plate compared to the TriFix. The difference for the screw tip cutout was +98% at 2000 cycles, +92% at 4000 cycles, and +65% at 6000 cycles for the SI plate compared to the TriFix.

In both groups, both the fracture gap-angle and screw tip cutout showed a significant increase over the number of cycles (all *p* ≤ 0.008). The TriFix showed a 3-fold increase in fracture gap angle and the SI Plate 3.5-fold between cycles 2000 and 6000. In the screw tip cutout, the TriFix increased 2.6-fold, and the SI-Plate increased 2.2-fold between cycles 2000 and 6000.

### 3.3. Number of Cycles to Failure

**Figure 7:**
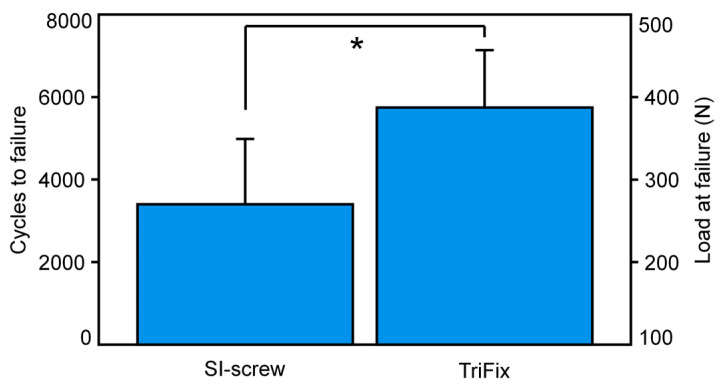
Mean number of cycles until failure with the standard deviation for both groups. Significant differences between the groups are marked with *.

The mean number of cycles to failure and the corresponding load at failure was 3399 cycles (SD 1583) and 270.0 N (SD 79.2 N) for the SI-screw, and 5747 cycles (SD 1389) and 387.4 N (SD 69.5 N) for the TriFix, respectively (Figure 7). This difference was statistically significant (*p* = 0.017). The TriFix showed a 69% increase in cycles to failure and a 44% increase in load to failure.

### 3.4. Mode of Catastrophic Failure

**Figure 8:**
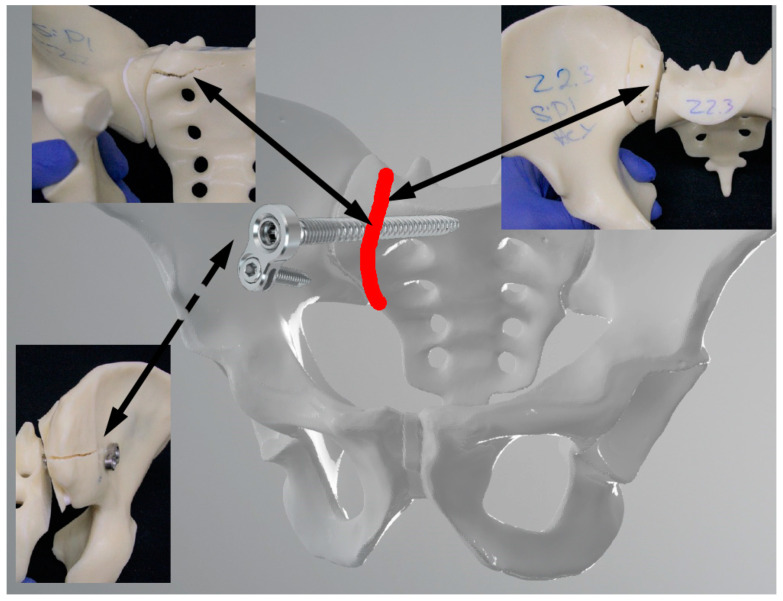
Failure pattern of the SI plate group projected onto a 3D pelvic model. The images show fractures of the sacrum and ilium and fracture gap dissociation. Red line = fracture line; dotted arrows indicate the location on the back of the model; sold arrows on the front.

**Figure 9:**
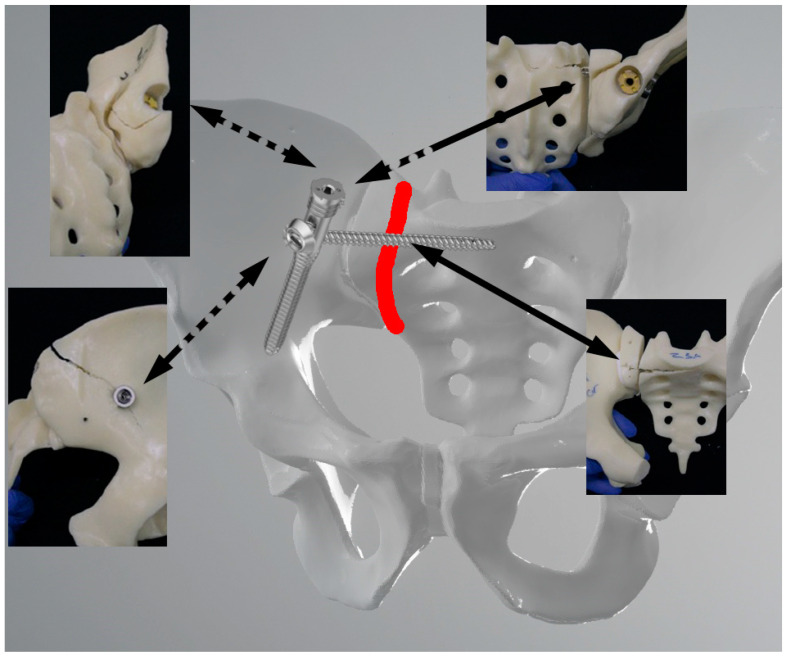
Failure pattern of the TriFix group projected onto a 3D pelvic model. The images show fractures of the sacrum and ilium, especially around the entry point of the ilium screw and fracture gap dissociation. Red line = fracture line; dotted arrows indicate the location on the back of the model; solid arrows on the front.

Figure 8 and Figure 9 show the catastrophic failure in the two groups. In addition to failing at the fracture plane, both groups also failed around the implants. In particular, fractures in the region of the entry points and implant trajectories were caused by the hard outer structure of the artificial bone material. 

## 4. Discussion

Increased life expectancy in recent decades has led to an increased incidence of fragility fractures of the pelvic ring [3,4,19]. The mechanisms of trauma and the resulting treatment differ from other types of pelvic ring fracture. One standard operative treatment is iliosacral screw osteosynthesis. However, in osteoporotic bone, single iliosacral screw fixation may be mechanically inadequate and carries a high risk of screw loosening [9,20].

For this reason, the present study investigates two advanced percutaneous fixation options for the fracture stabilization of the dorsal pelvic ring in a biomechanical setup. In this study, we were able to demonstrate superior biomechanical characteristics of the TriFix fixation consisting of a fenestrated iliac screw and an iliosacral screw with a quasi-angle stable connection compared to an iliosacral screw with an additional short locking screw. In biomechanical testing, implant loosening parameters and number of cycles to failure showed significantly superior results for the TriFix stabilization.

This allows the implant to be selected according to the biomechanical requirements of the fracture, instability, and bone morphology. In young patients with good bone quality and the ability to unload or partial weight bear their leg, a standard iliosacral screw is sufficient. However, if any factor changes, such as the ability to unload the leg or the bone quality, the addition of a short, angular stable iliac screw is an option to increase stability and prevent construct loosening. If the fracture is more unstable and the bone quality is poor, the TriFix can provide even more stability to help prevent complications.

Several previous studies have focused on improving implant anchorage, particularly in osteoporotic bone and unstable fracture patterns. Loosening of the screw in the sacrum and unthreading of the screw are the two main failure mechanisms of iliosacral screws in osteoporotic dorsal pelvic ring fractures. Therefore, iliosacral screw augmentation with bone cement is one method to reduce sacral screw loosening [15,21]. Cement augmentation significantly improved sacral screw fixation [22,23,24]. Oberkirchner et al. compared iliosacral screws with and without cement augmentation in a human pelvic model. In their pull-out tests, the augmented groups showed significantly higher primary stability compared to the non-augmented groups [10]. In order to increase patient safety, the screw augmentation technique was changed from injecting cement prior to screw placement to a clinically viable procedure using cannulated iliosacral screws with perforations at the tip, allowing cement to be injected after successful screw placement [15,21,25]. 

To address the issue of unscrewing, Zderic et al. developed the screw-in-screw prototype, which allows the additional placement of a short 2.7 mm locking screw in the ilium through a threaded hole in the iliosacral screw head [13]. Their biomechanical comparison of this prototype implant with standard iliosacral screws shows successful prevention of loosening but also greater biomechanical stability in terms of cycles to failure, screw flexion, cut-through, and screw tilt [13]. These results clearly demonstrate the significant biomechanical advantages of an additional short iliac locking screw over a standard iliosacral screw. Therefore, we decided not to include a standard iliosacral group in our study.

The biomechanical principle of the TriFix construct consists of an iliac screw, which acts as a reinforced fixation anchor in the iliac bone, and an iliosacral screw, which are both connected in a quasi-angle stable manner. Anchoring in the TriFix screw moves the anchor point medially, and the polyethylene inlay increases the contact surface between the implants, both of which contribute to improved construct stability. The TriFix screw is equivalent to a reinforced fixation anchor in the iliac bone. Therefore, failures such as the washer penetration described are virtually impossible [26]. 

To the best of our knowledge, there are no techniques described to improve iliosacral screw anchoring in the iliac bone. However, it is possible to augment the tip of the iliosacral screws presented with polymethacrylate through the existing perforations at the tip of the screw. These two features allow the use of the TriFix constructs in patients with extremely poor bone quality and unstable fractures. Furthermore, the modularity of this system allows for a quick and easy extension to spinopelvic stabilization if required [27]. 

Although the pelvic models used in this study mimicked osteoporotic bone structure, catastrophic failures were observed in both groups, which are not known from the clinical situation and underline the strength of both constructs presented. However, the stability of the TriFix screw construct was significantly higher, with up to 70% more load cycles to failure compared to the SI plate group. 

While implantation of the SI-Plate is mainly comparable to standard iliosacral screws, which can be performed in the supine or prone position, implantation of the TriFix screws requires the patient to be in the prone position. Surgeons may have to adapt to a new patient position, which may be seen as a disadvantage in the clinical setting. In our experience, the prone position is ideal for screw osteosynthesis of the dorsal, pelvic ring unless a supraacetabular external fixator is unavoidable. The clinical advantages of the implants used, particularly in terms of handling, have already been published [27].

There are also limitations to this study. An artificial pelvis model does not show physiological behavior, as mentioned above. In particular, the insertion of the iliac screw in the TriFix group differed from the clinical situation due to the brittle nature of the cortical bone. A study using cadaveric specimens may give an even more reliable result from a clinical point of view but would have the disadvantage of reduced comparability between the specimens used and the type of instrumentation due to different anatomical aspects and bone properties. However, several biomechanical studies were carried out using these bone models, allowing comparison of results between studies [12,13]. Therefore, we decided to use this osteoporotic artificial pelvis model. Another critical aspect is biomechanical testing, which can only investigate initial stability. However, cyclic loading is more informative than static failure testing. The setup used is comparable to several previous biomechanical studies and also allows for comparison of results, which is an advantage of the tests performed [12,13].

Clinical trials should be the next step in confirming the results of this preclinical biomechanical study.

## 5. Conclusions

Our results show that the primary stability and implant anchorage of osteosynthesis of the dorsal pelvic ring can be increased using the triangular fixation system and that the stiffness does not differ between the triangular fixation system and the SI plate group. Therefore, we conclude from the results of our biomechanical study that the use of the triangular fixation system has advantages, especially in weak bones and/or unstable fractures, which need to be confirmed in clinical trials.

## Data Availability

All data are available on request from the corresponding author.

## References

[1] Audretsch C.K., Siegemund A., Ellmerer A., Herath S.C. (2023). Sex Differences in Pelvic Fractures-a Retrospective Analysis of 16 359 Cases From the German Trauma Registry. Dtsch. Ärzteblatt Int..

[2] Rommens P.M., Hofmann A. (2023). The FFP-classification: From eminence to evidence. Injury.

[3] Rollmann M.F., Herath S.C., Kirchhoff F., Braun B.J., Holstein J.H., Pohlemann T., Menger M.D., Histing T. (2017). Pelvic ring fractures in the elderly now and then—A pelvic registry study. Arch. Gerontol. Geriatr..

[4] Fuchs T., Rottbeck U., Hofbauer V., Raschke M., Stange R. (2011). Beckenringfrakturen im Alter. Der Unfallchirurg.

[5] Rommens P.M., Hofmann A., Kraemer S., Kisilak M., Boudissa M., Wagner D. (2022). Operative treatment of fragility fractures of the pelvis: A critical analysis of 140 patients. Eur. J. Trauma Emerg. Surg..

[6] Maier G.S., Kolbow K., Lazovic D., Horas K., Roth K.E., Seeger J.B., Maus U. (2016). Risk factors for pelvic insufficiency fractures and outcome after conservative therapy. Arch. Gerontol. Geriatr..

[7] Bukata S.V., Digiovanni B.F., Friedman S.M., Hoyen H., Kates A., Kates S.L., Mears S.C., Mendelson D.A., Serna F.H., Sieber F.E. (2011). A guide to improving the care of patients with fragility fractures. Geriatr. Orthop. Surg. Rehabil..

[8] Hopf J.C., Krieglstein C.F., Müller L.P., Koslowsky T.C. (2015). Percutaneous iliosacral screw fixation after osteoporotic posterior ring fractures of the pelvis reduces pain significantly in elderly patients. Injury.

[9] Rommens P.M., Hofmann A. (2013). Comprehensive classification of fragility fractures of the pelvic ring: Recommendations for surgical treatment. Injury.

[10] Oberkircher L., Masaeli A., Bliemel C., Debus F., Ruchholtz S., Krüger A. (2016). Primary stability of three different iliosacral screw fixation techniques in osteoporotic cadaver specimens-a biomechanical investigation. Spine J..

[11] Grechenig S., Gansslen A., Gueorguiev B., Berner A., Muller M., Nerlich M., Schmitz P. (2015). PMMA-augmented SI screw: A biomechanical analysis of stiffness and pull-out force in a matched paired human cadaveric model. Injury.

[12] Lodde M.F., Katthagen J.C., Schopper C.O., Zderic I., Richards G., Gueorguiev B., Raschke M.J., Hartensuer R. (2021). Biomechanical Comparison of Five Fixation Techniques for Unstable Fragility Fractures of the Pelvic Ring. J. Clin. Med..

[13] Zderic I., Wagner D., Schopper C., Lodde M., Richards G., Gueorguiev B., Rommens P., Acklin Y.P. (2021). Screw-in-screw fixation of fragility sacrum fractures provides high stability without loosening-biomechanical evaluation of a new concept. J. Orthop. Res..

[14] Guerin G., Laghmouche N., Moreau P.E., Upex P., Jouffroy P., Riouallon G. (2020). Iliosacral screwing under navigation control: Technical note. Orthop. Traumatol. Surg. Res..

[15] König M.A., Hediger S., Schmitt J.W., Jentzsch T., Sprengel K., Werner C.M.L. (2018). In-screw cement augmentation for iliosacral screw fixation in posterior ring pathologies with insufficient bone stock. Eur. J. Trauma Emerg. Surg..

[16] Ellmerer A.E., Küper M.A., Rollmann M.F., Herath S.C., Histing T. (2022). Cement augmentation in pelvic ring fractures. Unfallchirurgie.

[17] Suero E.M., Greiner A., Becker C.A., Cavalcanti Kussmaul A., Weidert S., Pfeufer D., Woiczinski M., Braun C., Flatz W., Bocker W. (2021). Biomechanical stability of sacroiliac screw osteosynthesis with and without cement augmentation. Injury.

[18] Chakkravarthy V., Manojkumar P., Lakshmanan M., Eswar Prasad K., Dafale R., Vadhana V.C., Narayan R.L. (2023). Comparing bio-tribocorrosion of selective laser melted Titanium-25% Niobium and conventionally manufactured Ti-6Al-4 V in inflammatory conditions. J. Alloys Compd..

[19] Oberkircher L., Ruchholtz S., Rommens P.M., Hofmann A., Bucking B., Kruger A. (2018). Osteoporotic Pelvic Fractures. Dtsch. Ärzteblatt Int..

[20] Dudda M., Hoffmann M., Schildhauer T.A. (2013). Sakrumfrakturen und lumbopelvine Instabilitäten bei Beckenringverletzungen. Der Unfallchirurg.

[21] Höch A., Pieroh P., Henkelmann R., Josten C., Böhme J. (2017). In-screw polymethylmethacrylate-augmented sacroiliac screw for the treatment of fragility fractures of the pelvis: A prospective, observational study with 1-year follow-up. BMC Surg..

[22] König A., Oberkircher L., Beeres F.J.P., Babst R., Ruchholtz S., Link B.C. (2019). Cement augmentation of sacroiliac screws in fragility fractures of the pelvic ring-A synopsis and systematic review of the current literature. Injury.

[23] Osterhoff G., Dodd A.E., Unno F., Wong A., Amiri S., Lefaivre K.A., Guy P. (2016). Cement Augmentation in Sacroiliac Screw Fixation Offers Modest Biomechanical Advantages in a Cadaver Model. Clin. Orthop. Relat. Res..

[24] Collinge C.A., Crist B.D. (2016). Combined Percutaneous Iliosacral Screw Fixation With Sacroplasty Using Resorbable Calcium Phosphate Cement for Osteoporotic Pelvic Fractures Requiring Surgery. J. Orthop. Trauma.

[25] Hack J., Krüger A., Masaeli A., Aigner R., Ruchholtz S., Oberkircher L. (2018). Cement-augmented sacroiliac screw fixation with cannulated versus perforated screws—A biomechanical study in an osteoporotic hemipelvis model. Injury.

[26] Crist B.D., Pfeiffer F.M., Khazzam M.S., Kueny R.A., Della Rocca G.J., Carson W.L. (2019). Biomechanical evaluation of location and mode of failure in three screw fixations for a comminuted transforaminal sacral fracture model. J. Orthop. Translat..

[27] Gruneweller N., Wahnert D., Vordemvenne T. (2021). Instability of the posterior pelvic ring: Introduction of innovative implants. J. Orthop. Surg. Res..

